# Catalytic Reduction of Carbon Dioxide on the (001), (011), and (111) Surfaces of TiC and ZrC: A Computational Study

**DOI:** 10.1021/acs.jpcc.1c10180

**Published:** 2022-03-14

**Authors:** Fabrizio Silveri, Matthew G. Quesne, Francesc Viñes, Francesc Illas, C. Richard A. Catlow, Nora H. de Leeuw

**Affiliations:** † School of Chemistry, Cardiff University, Main Building, Park Place, Cardiff CF10 3AT, U.K.; ‡ Departament de Ciència de Materials i Química Física and Institut de Química Teòrica i Computacional (IQTCUB), Universitat de Barcelona, c/ Martí i Franquès 1-11, 08028 Barcelona, Spain; § Gemmate Technologies s.r.l., via Reano, 31, 10090 Buttigliera Alta, TO, Italy; ∥ UK Catalysis Hub, Research Complex at Harwell, STFC Rutherford Appleton Laboratory, Didcot, Oxfordshire OX11 0FA, U.K.; ⊥ Department of Chemistry, University College London, 20 Gordon Street, London WC1 HOAJ, U.K.; # School of Chemistry, University of Leeds, Leeds LS2 9JT, U.K.

## Abstract

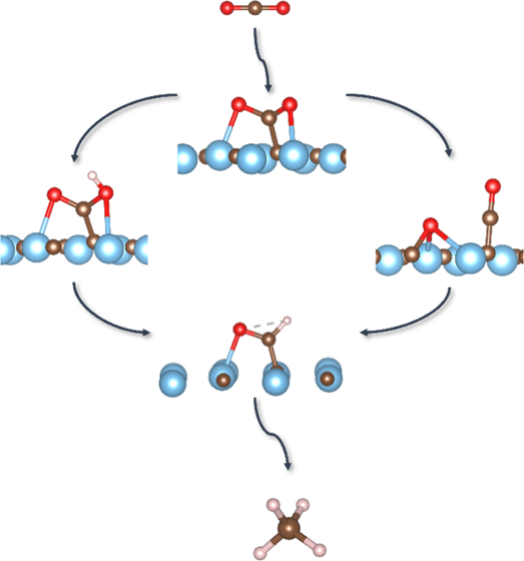

We present a computational study of the activity and selectivity of early transition-metal carbides as carbon dioxide reduction catalysts. We analyze the effects of the adsorption of CO_2_ and H_2_ on the (001), (011), and metal-terminated (111) surfaces of TiC and ZrC, as carbon dioxide undergoes either dissociation to CO or hydrogenation to COOH or HCOO. The relative stabilities of the three reduction intermediates and the activation energies for their formation allow the identification of favored pathways on each surface, which are examined as they lead to the release of CO, HCOOH, CH_3_OH, and CH_4_, thereby also characterizing the activity and selectivity of the two materials. Reaction energetics implicate HCO as the key common intermediate on all surfaces studied and rule out the release of formaldehyde. Surface hydroxylation is shown to be highly selective toward methane production as the formation of methanol is hindered on all surfaces by its barrierless conversion to CO.

## Introduction

The growing awareness and impact of climate change have led to ambitious sustainable development goals in all sectors of the industry. Carbon capture and utilization schemes are one of the many routes that have been proposed to increase the environmental sustainability of the transport and chemical industries. The aim of this strategy is to close the cycle of fossil fuel burning by using renewable energy to recycle water and carbon dioxide into fuels and chemicals, thereby mimicking the outcome of natural photosynthesis.^1,2^

The synthetic fixation of CO_2_ into fuels and chemicals can be performed through two main strategies: by sequestering CO_2_ into existing molecular frameworks without changing its oxidation state or by reducing the CO_2_ into base chemicals that can be further processed. While the former strategy has already been implemented in several industrial processes as a means of reducing the environmental impact of an industrial process by recycling the emitted CO_2_, its use is limited by the demand for chemicals that can be produced through either exothermic or mildly endothermic reactions—typically urea, carbonates, and polymers^3^—which are several orders of magnitude lower in carbon density than the global carbon emissions.^4^ In contrast, direct carbon dioxide reduction with water or other readily available natural chemicals is a highly endothermic process, requiring either large amounts of energy or the use of highly reductive chemicals to obtain the desired product. Several processes have been proposed for CO_2_ recycling using the oxidation of renewably generated H_2_ to provide the energy required for the reaction, which often makes use of technology that is already employed by the chemical industry. Target chemicals for CO_2_ reduction can be CO, HCOOH, CH_3_OH, and CH_4_, which can either be used as fuels or transformed into a variety of fuels and chemicals using the technology currently employed by the chemical industry.^1,5^ The reduction reactions, however, often produce a mixture of these and other carbon-based products, making selectivity an issue that must be tackled as much as activity, since the separation of chemicals can be a complex and expensive process.^6^ For this reason, several studies have investigated the product selectivity of CO_2_ reduction over various catalysts, and theoretical effort has often focused on this aspect of the catalytic reduction to provide insight into the design of suitable catalysts.^7−9^

The computational investigation of the catalytic activity of CO_2_ reduction over Mo_2_S was able to correlate its remarkable selectivity toward CO (with a ratio of 154:1 to CH_3_OH), with its computed oxygen binding energy.^10^ In addition, adsorption studies were performed on copper catalysts with metal oxide support, predicting higher activity at the interface between the two materials as a result of the preferential binding of H_2_ on the metal, while CO_2_ favors adsorption on the oxide.^6^ The effect of oxygen vacancies and surface morphology on carbon dioxide activation has been highlighted by similar studies on Cu_2_O, In_2_O_3_, and TiO_2_.^11^ The reaction pathway has been investigated in several studies, identifying two recurring processes, which are selected by the site of first hydrogenation of CO_2_: if this is on the carbon atom, producing an HCOO species on the catalyst surface, the first step is rate-determining and the catalyst cannot produce CO, as the product will necessarily be hydrogenated; however, if the first hydrogenation involves the oxygen, the selectivity will depend on the relative rates of CO desorption and CO hydrogenation, as the latter will be the rate-determining step for methanol formation.^12^ In some cases, the mechanism was reported to change depending on the surface stoichiometry of the material, e.g., molybdenum carbides, for which combined computational and experimental studies have highlighted how selectivity toward either CO or CH_3_OH is driven through a complex set of reactions by the carbon/metal ratio of the exposed surface.^13^ Transition-metal carbides of groups 4–6 have been investigated both experimentally and computationally for CO_2_ reduction, giving promising results. However, less work has been dedicated to group 4 transition-metal carbides. Porosoff et al.^10^ have shown low activity for TiC compared to that of MoC and Mo_2_C, linking that to a high oxygen binding energy, which hinders the catalytic cycle on TiC surfaces. Nevertheless, Quesne et al.^14,15^ obtained interesting work function and d-band center values for TiC and ZrC surfaces, which can be correlated with the surface ability to catalyze reduction reactions. Moreover, CO_2_ shows significant bond elongation and charge transfer upon adsorption on these materials, suggesting the possibility of catalyzing a reduction reaction.

In this study, TiC and ZrC were therefore chosen for a thorough investigation of their catalytic activity toward CO_2_ dissociation and hydrogenation upon their three lowest Miller index surfaces. The investigation was carried out through the density functional theory (DFT) modeling of each of the (001), (011), and metal-terminated (111) surfaces of the two materials, with the aim of identifying the most energetically efficient pathways for the formation of CO, HCOOH, CH_3_OH, and CH_4_ from carbon dioxide and molecular hydrogen and to identify the critical factors controlling their activity and selectivity. The two carbides and their respective surfaces were identified following previous work on groups 4 and 5 transition metals, which gave promising results for surface properties as well as for CO_2_ and H_2_ adsorption,^14−16^ despite the high surface energy of the (111) surfaces.^14,17^

## Computational Methods

All calculations reported in this work were performed within the framework of the periodic density functional theory using the VASP code (Vienna ab initio software package) version 5.4.1.^18^ Core electrons were described using the projected-augmented wave (PAW) method,^19^ whereas a plane-wave basis set has been used to expand the valence electron density. The Perdew–Burke–Ernzerhof (PBE) functional^20^ was used to approximate exchange and correlation interactions in the framework of generalized gradient approximation (GGA). Additionally, long-range-dispersive interactions were modeled using the Grimme D3 dispersion method.^21^ All energies are converged within a cutoff of 520 eV and an electronic self-consistent field (SCF) threshold of 10^–5^ eV. Convergence was determined using the tetrahedron method, implementing Blochl corrected smearing.^22^ A dipole correction was enabled in all directions to avoid numerical problems with the perpendicular dipole inherent in the (111) facets. All parameters were benchmarked to optimize computational time and accuracy for the calculations.

The surfaces of the transition-metal carbides (TMCs) studied in the present work were simulated by six atomic layers within (2 × 2) supercell slab models, cutting the bulk along the (001), (011), and (111) planes, as previously reported by Quesne et al.^14^ Hence, each slab surface model has 6 atomic layers and 16 atoms per layer. The (001) and (011) surfaces are created so that they preserve bulk stoichiometry, resulting in an equal number of carbon and metal atoms being exposed to the vacuum. Conversely, the (111) plane is parallel to carbon and metal atomic layers, resulting in two possible surface terminations that, respectively, expose carbon and metal atoms to the vacuum in such a way that the stoichiometry is preserved. Such a protocol has been previously applied to TMCs and has been shown to describe accurately the electronic and structural properties of the (111) surfaces and is consistent with previous reports of the same materials.^14^ To avoid interactions along the axis perpendicular to the surface, the lattice parameter was increased by 12 Å in such a direction. A 5 × 5 × 1 **k**-points reciprocal lattice matrix was generated using the Monkhorst–Pack method. These parameters were benchmarked, ensuring that vacuum spacing, grid **k**-points, and cutoff energy values allowed convergence to a constant value of the energy. Spin polarization was allowed for the determination of the energy of all structures except for the H_2_ reference molecule. Atomic charges were calculated for specific systems through a Bader charge analysis. The energy of the system was minimized by keeping the cell parameters fixed at their bulk-optimized value and allowing relaxation of all atoms excepting those belonging to the two bottom layers of the model slab, which were kept fixed. The minimum energy structures were found using a built-in DIIS algorithm with a convergence force threshold of 10^–2^ eV/Å. The energies of molecular H_2_, CO_2_, CO, O_2_, HCOH, HCOOH, CH_3_OH, and CH_4_ have been used as references for the adsorption and desorption energies on transition-metal carbide slabs, using half of the energy of the H_2_ and O_2_ molecules as a reference for atomic H and O adsorption. These calculations have been performed using a single **Γ k**-point and the same cutoff energy as all other calculations within a suitable unit cell, optimized so as to minimize the interaction between neighboring images.

The electronic energies thus calculated were used to compute the reaction and activation energies of the reaction path from CO_2_ to its reduced products, which we consider sufficient to inform our understanding of the mechanistic aspects, which is the main objective of this study. Transition-state structures were primarily located using the built-in CI-NEB (climbing image nudged elastic band) algorithm of the VASP code; these calculations used a variable number of system images, in the range 8−13, connected by springs, and was usually performed in two steps: the first was to find an initial guess of the minimum energy path between reactants and products by relaxing the full string of connected images, while the second aimed at identifying the saddle point by turning on the climbing image algorithm on the highest energy image. In the few cases in which the saddle point of the potential energy surface (PES) proved difficult to isolate using this method, an additional third step was used, employing the built-in Dimer method algorithm on the closest NEB image to obtain the relevant transition-state structure. All presented transition-state structures were confirmed to be PES saddle points through a vibrational frequency calculation limited to the adsorbed species and their respective adsorption sites on the surface.

In analyzing the reduction path of CO_2_, the key quantities are the adsorption energy (*E*_ads_), overall reaction energy (*E*_r_), reduction step energy (*E*_s_), and the activation energy (*E*_a_), which are defined as follows:

Adsorption energy (*E*_ads_):
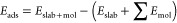
1The adsorption energy is defined in terms of *E*_slab+mol_, the total electronic energy of an adsorbed chemical species on a surface, *E*_slab_, the energy of the bare surface, and *E*_mol_, the electronic energy of the respective isolated molecule(s).

Overall reaction energy (*E*_r_):
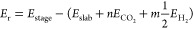
2The overall reaction energy of stage *n* is defined as the difference between its total electronic energy (*E*_stage_) and the sum of the electronic energies of its respective surface and gas-phase molecular reactants in terms of CO_2_ and H_2_.

Single-step energy (*E*_s_):
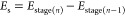
3The reduction step energy of each stage is instead the energy difference between two subsequent intermediates or products along any reduction path.

Activation energy (*E*_a_):
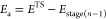
4For a given elementary step, the activation energy is the total energy difference between the identified transition-state structure and the previous stable intermediate.

Each one of the reaction steps and transition states reported in this paper is the result of geometrical optimizations which included distinct initial geometries or reaction coordinates, so that the minimum energy products and reaction paths could be identified for each investigated step. In the case of hydrogenation reactions, molecular hydrogen was always considered to be dissociatively adsorbed on the catalytic surfaces prior to the reaction, consistently with a Langmuir–Hinshelwood mechanism. This choice is consistent with the results of previous works,^16,23^ which highlighted small or nonexistent activation barriers for hydrogen chemisorption on the TiC(001) surface and even greater chemical driving force for dissociative adsorption on all other investigated surfaces. Other hydrogenation mechanisms were therefore not investigated.

## Results and Discussion

The investigation of the CO_2_ reduction on TiC and ZrC surfaces was carried out in two steps. Initially, the first CO_2_ reduction step is analyzed through the three reaction pathways leading to adsorbed CO + O, COOH, and HCOO; the reaction and activation energies of the three reactions are investigated on each of the six surfaces, and the most favorable reaction pathways are identified on each surface. Subsequently, the identified pathways are investigated until the final reduced products, highlighting the activity and selectivity of each surface for the conversion of CO_2_ to CO, HCOOH, CH_3_OH, and CH_4_.

### Oxidized and Reduced Carbon on TiC and ZrC Surfaces

**Figure 1:**
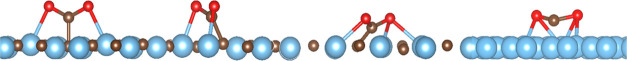
CO_2_ adsorption sites on TiC. From left to right: C_on-top_ on (001), M_2_C on (001), M_4_C on (011), and M_5_ on (111). The same adsorption sites are also found on ZrC(001). The color scheme is as follows: Ti, blue; C, brown; and O, red.

The electronic and geometrical effects of CO_2_ adsorption are very similar on TiC and ZrC. All surfaces of TiC and ZrC show a favorable chemisorption of CO_2_. The molecule is strongly bound to a surface carbon atom on (001) and (011) surfaces, on which all results are comparable to the previously reported computational work on the same surfaces^15^ and show similar adsorption patterns as those highlighted on Ti_2_C MXenes.^24,25^ The main CO_2_ adsorption geometries identified for these surfaces, C_on-top_ for (001) surfaces and M_2_C and M_4_C on (011) surfaces, are displayed in Figure 1. On the two (111) surfaces, however, we find an adsorption geometry that has not been previously reported: upon adsorption, the CO_2_ molecular plane is almost parallel to the plane of the surface, with the two oxygen atoms coordinating 5 metal atoms in the M_5_ adsorption site. Longer bond lengths and more reduced CO_2_ molecules are found, as the bond elongates up to +0.3 Å compared to the gas-phase molecule and the CO_2_ carbon atom gains +1.8 *e* of charge, but the adsorption is less exothermic than on the (011) surfaces, as *E*_ads_ is computed to be of −3.1 eV on TiC(111) and of −3.2 eV on ZrC(111). Previous studies^15^ only reported vertical adsorption sites for these surfaces, which show significantly less exothermic adsorption. As can be seen in Figure 1, all four identified adsorption sites have a bent geometry.

**Figure 2:**
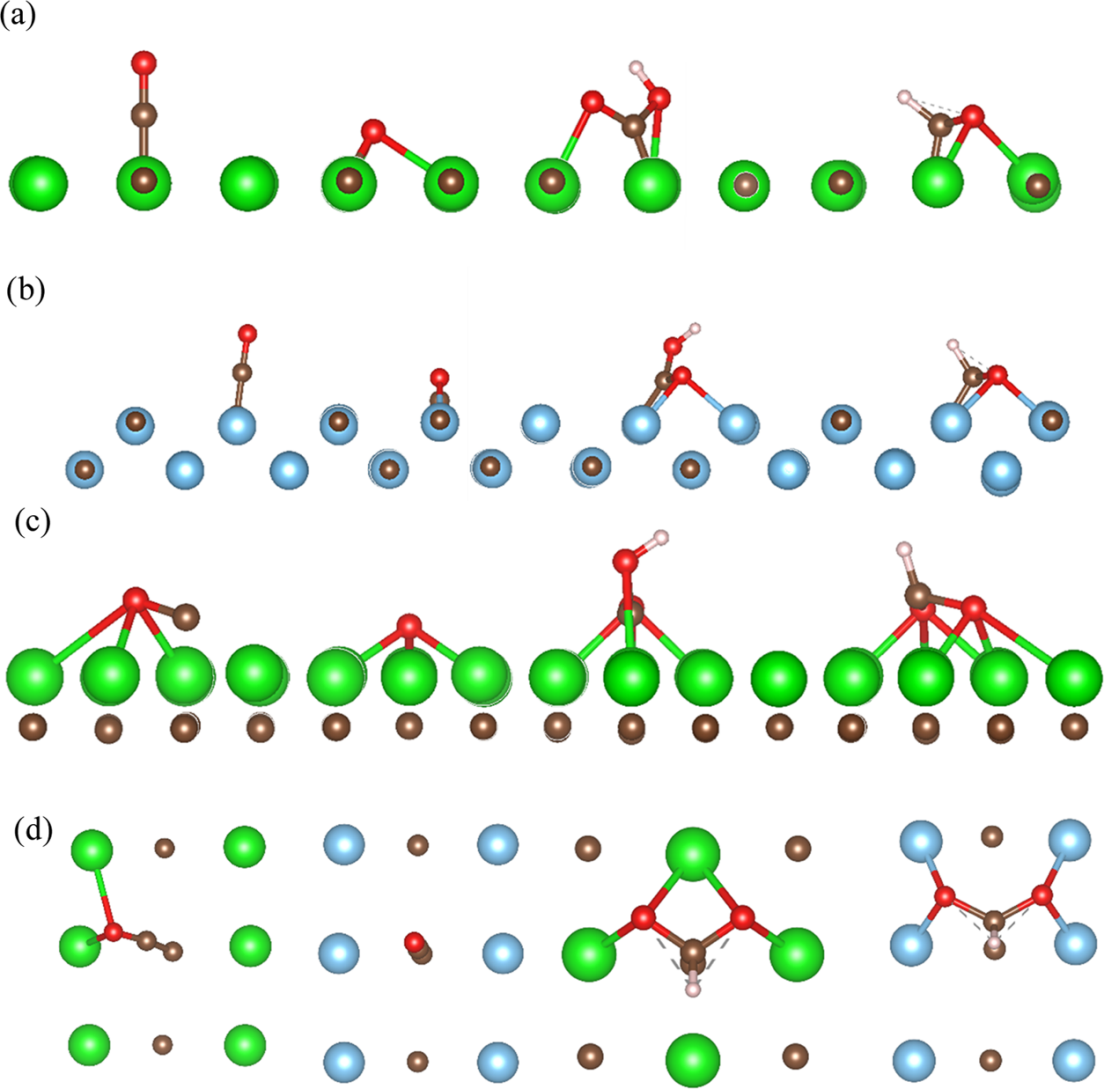
Most favorable adsorption geometries of, from left to right, CO, O, COOH, and HCOO on (a) ZrC(001), (b) TiC(011), and (c) Zr-terminated ZrC(111). (d) Top view, added for clarity, of (from left to right) CO on TiC(011), CO on ZrC(011), HCOO on ZrC(001), and HCOO on TiC(011). Analogous geometries are found for all species not shown in this picture, except for HCOO on ZrC(011), which spontaneously dissociates upon adsorption. The adsorption geometries shown here are those with the minimum energy among those tested on each surface. The color scheme is as follows: Ti, blue; Zr, green; C, brown; O, red; and H, pink.

In the initial step of the catalytic reduction process, CO_2_ may either dissociate yielding CO and O on the surface of the carbide or be hydrogenated at the C or O atoms, yielding, respectively, a carboxyl or a formate on the catalyst surface. In the latter case, the hydrogen atom is transferred from the surface of the catalyst since the dissociation of the H_2_ molecule has previously been shown to be spontaneous in all but the most unfavorable conditions.^16^ These first reaction steps have been investigated on all surfaces, modeling the formation of the three first-step intermediates
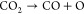
a
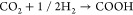
b
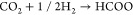
cThe adsorption of each of the chemical species on every surface has therefore been studied, identifying the minimum energy structures shown in Figure 2 and highlighting the adsorption energies reported in Table 1. The adsorption of CO is generally very exothermic (*E*_ads_ = −1.82 to −3.91 eV) on all surfaces. C_on-top_ sites are dominant on both (001) surfaces, as shown by Figure 2a, in which the molecule stands almost perpendicular to the plane of the catalyst, only slightly leaning toward a metal atom; on these sites, the C–O bond shows significant elongation (increasing to 1.20 Å), as the distance between the adsorbed molecule and the nearest surface carbon atom also resembles that of a strong covalent C–C bond at 1.32 Å. The (011) surfaces lead to the most exothermic CO adsorption on both carbides, as was previously noted for the case of CO_2_; Figure 2b shows the most favorable adsorption site on TiC(001), where the CO molecule is roughly perpendicular to the surface and coordinated only through the carbon atom. While a similar adsorption is also possible on ZrC(011), a more exothermic adsorption site is present, in which the CO molecule is roughly parallel to the surface and coordinates a surface carbon with its carbon atom and two metal atoms with is oxygen atom; on both surfaces, the molecule appears to be activated, as shown by the elongation of the C–O bond, which reaches 1.27 Å on ZrC(011). Both adsorption sites are shown in Figure 2d.

**Table 1:** Adsorption Energies for CO, O, COOH, and HCOO on TiC and ZrC, Given in eV^a^

	CO	O	COOH	HCOO
TiC				
(001)	–1.82 (C_on-top_)	–2.54 (M_2_C)	–1.25 (M_2_C)	–2.08 (C_on-top_)
(011)	–3.22 (C_on-top_)	–4.40 (M-O-C)	–1.82 (M_4_C)	–5.35 (M_4_C)
(111)	–3.22 (M_2_)	–5.76 (M_3_)	–1.20 (M_4-vertical_)	–6.12 (M_5-up_)
ZrC				
(001)	–2.13 (C_on-top_)	–2.79 (M_2_C)	–1.70 (M_2_C)	–3.29 (C_on-top_)
(011)	–3.91 (C-M_bridge_)	–4.56 (M_2-bridge_)	–3.74 (C_on-top_)	N/A
(111)	–3.13 (M_2_)	–5.85 (M_3_)	–2.46 (M_4-vertical_)	–6.17 (M_5-up_)

a Only the most favorable adsorption sites are considered in this table.

The (111) surfaces offer multiple coordination patterns, resulting in low energy structures where both atoms of CO maximize coordination by lying almost parallel to the surface, as shown in Figure 2c. Isolated oxygen atoms are bound very strongly to all surfaces (*E*_ads_ = −2.5 to −5.9 eV), as expected from the well-known tendency of TMCs to form oxycarbides.^26^ On the (001) surfaces, adsorbed O is coordinated to one carbon and two metal atoms. On the (011), oxygen interacts very strongly with carbon atoms, pulling them strongly out of the plane of the surface, whereas on the (111), O fits in the hexagonal sites coordinating three metal atoms, binding strongly to the surface.

The carboxyl group formed by the hydrogenation of CO_2_ on one of the oxygen atoms also has different optimized geometries on the different surfaces. On both (001) surfaces, two planar structures could be identified: the first, named as C_on-top_ adsorption site, has its O–C–O plane perpendicular to the surface and is slightly bent to maximize the interaction between the nonhydrogenated oxygen and the surface; alternatively, the second structure, the M_2_C adsorption site, is bent toward the surface to maximize the interaction between the latter and the oxygen atoms. The two structures have very similar energies (Δ*E*_ads_ < 0.05 eV), with the latter at a slightly lower energy, which is shown in Figure 2a. On the (011), similar structures are found albeit with different relative stabilities, with C_on-top_ being stable on ZrC, while the M_2_C adsorption site is favored on TiC. On the (111) surface of both carbides, the COOH group is most stable when aligned perpendicularly to the surface, with C=O coordinated to the surface and −OH pointing upwards.

Formate groups formed by the hydrogenation of a CO_2_ carbon atom are not stable on ZrC(011), giving spontaneous dissociation to an sp^2^-hybridized HCO on a C_on-top_ site, with O coordinated to a metal, plus adsorbed oxygen. The same process was expected for TiC(011), but in this case, a surface formate intermediate could be isolated, as shown in Figure 2b. On the four other surfaces, HCOO has stable configurations similar to those for COOH, with C_on-top_ and M_5_ being the most favored on (001) and (111) surfaces, respectively, on both carbides.

Across the three surfaces, adsorption shows similar characteristics for TiC and ZrC, as adsorption geometries and energies are similar on the two materials for the (001) and (111) surfaces, where adsorption geometries are always equivalent, with only minor differences due to the change in lattice parameters between the two cells. In contrast, all adsorption sites identified for the (011) surfaces differ between the two materials. Energetically, adsorption on ZrC is always shown to be more exothermic than the equivalent adsorption on TiC; the energy difference is usually small (0.02–0.35 eV) but exceeds 1 eV for HCOO/(001), COOH/(011), and COOH/(111).

### CO_2_ Dissociation and Hydrogenation

As we have seen, the species discussed above are all adsorbed exothermically. However, when we consider the energetics of reactions 1–3 above, we find that the energy of the reaction step on the surface is only negative for the dissociation reactions, while it can be significantly positive for the two hydrogenation pathways.

Table 2 summarizes the reaction energies associated with the formation of CO, COOH, and HCOO, reported with respect to gas-phase CO_2_ (+ 1/2 H_2_ in the case of hydrogenation) and to the single reaction step from adsorbed CO_2_. As noted, the dissociation reactions are the only ones for which single-step energies are negative, due to the high adsorption energies found for CO and O, which bind strongly to the surfaces of TiC and ZrC. However, previous reports^8,10^ have shown that when oxygen is bound too strongly, it can hinder the catalytic cycle. In contrast, single-step reaction energies for the formation of COOH and HCOO are generally endothermic, as a result of the relative instability of these intermediates. Nevertheless, the reaction energies from gas-phase reactants are generally exothermic, the formation of HCOO on the TiC(001) surface being the only exception, for which *E*_r_ = +0.19 eV. As a result, it is possible that the adsorption of CO_2_ and H_2_ on the low-index surfaces of TiC and ZrC might provide the energy for the formation of these intermediates on the catalysts, especially if an Eley–Rideal type of mechanism is operative.^27^ The endothermicity of CO_2_ hydrogenation can also be linked to the results of hydrogen adsorption on TMCs^16^ since hydrogenation on TiC and ZrC surfaces is always exothermic but energy is required for the formation of HCOO and COOH, as one strongly bound H atom has to be removed from binding directly to the surface. On the (011) and (111) surfaces, because of their more exothermic adsorptions, this results in especially high single-step energies for COOH formation. HCOO formation, however, is less thermodynamically unfavorable on the (111) surfaces, owing to the very strong bond between this intermediate and the surface.

**Table 2:** Reaction Energies and Single-Step Energies (in eV) for the Dissociation to CO + O, Hydrogenation to COOH, and Hydrogenation to HCOO^a^

	CO + O		COOH		HCOO	
	*E*_r_	*E*_s_	*E*_r_	*E*_s_	*E*_r_	*E*_s_
TiC						
(001)	–1.13	–0.23	–1.25	0.59	0.19	2.03
(011)	–7.05	–3.6	–3.38	1.43	–3.38	1.43
(111)	–5.70	–2.60	–2.76	1.43	–3.84	0.35
ZrC						
(001)	–1.72	–0.04	–1.70	0.95	–1.02	1.63
(011)	–5.03	–0.84	–3.73	1.71	–5.92*	–0.48*
(111)	–5.67	–2.49	–2.47	1.91	–3.9	0.48

a For each reaction, two energies are reported, differing for the reference used: in the column on the left of each section, the reaction energy *E*_r_ is reported, which is the energy of the intermediate on the surface of the catalyst minus the energy of the gas-phase reactants and pristine surface; in the column on the right of each section, the single-step energy *E*_s_ is reported, which is the energy of the intermediate on the surface of the catalyst minus the energy of the previous intermediate on the same surface. The energies reported for HCOO on ZrC(011) are marked with an asterisk because they are referred to the formation of HCO + O since HCOO dissociation occurs spontaneously on this surface.

From the results of Table 2, it is also possible to make a preliminary assessment of how the three competitive reactions are balanced on each surface. In absolute terms, the dissociation is always thermodynamically favored, as it always shows *E*_s_ < 0 for the single-step process. However, if only the hydrogenation reactions are considered, a strong preference toward the formation of COOH is found on (001) surfaces, while on (111) surfaces, the preference is toward the formation of HCOO, which on TiC(111) has the least positive single-step reaction energy of all hydrogenated intermediates. On the two (011) surfaces, the formate is unstable and can dissociate spontaneously to CHO + O. It was possible to identify a formate intermediate on TiC(011), for which *E*_s_ = 1.43 eV, the same as for CO_2_ hydroxylation. On ZrC(011), however, HCOO is so unstable that it undergoes spontaneous dissociation and no intermediate could be found. Table 2 reports *E*_s_ = −1.48 eV for this reaction, which refers to the formation of CHO + O.

**Figure 3:**
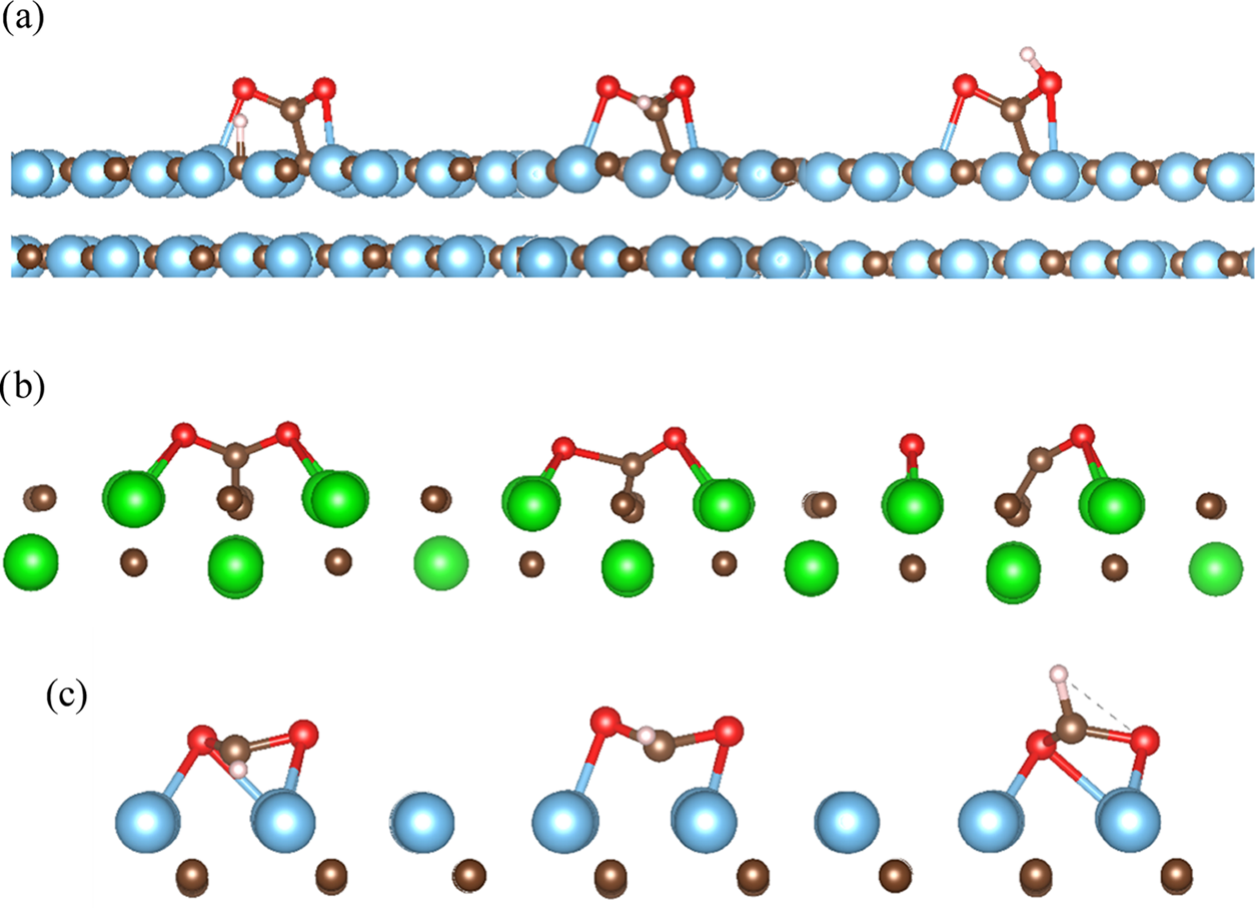
Proposed reduction mechanism of (a) CO_2_ hydrogenation to COOH on TiC(001), (b) CO_2_ dissociation to CO + O on ZrC(011), and (c) CO_2_ hydrogenation to HCOO on TiC(111). From left to right: initial state, in which the reactants are adsorbed on the surface of the catalyst; transition state, identified through the presence of one imaginary frequency related to the formation of the desired product; and final state, in which the product is adsorbed on the surface of the reactant. The color scheme is as follows: Ti, blue; Zr, green; C, brown; O, red; and H, pink.

To evaluate the selectivity of each surface, the simple prediction of reaction energies is insufficient. Nudged elastic band calculations (NEB and CI-NEB) and the dimer method have been employed to sample the region of the potential energy surface connecting each intermediate to their adsorbed reactants, identifying the transition states corresponding to the reactions leading to CO, COOH, and HCOO on (001), (011), and (111) surfaces. From this investigation, two reactions have been excluded: the formation of formate on TiC(001) and ZrC(001) has been considered too high in energy to take place, the former showing the only positive reaction energy from gas-phase reactants, the latter being strongly unfavorable compared to COOH formation. Figure 3 illustrates the initial state, transition state, and final state of the PES linking adsorbed CO_2_ and H to COOH on TiC(001), CO_2_ to CO and O on ZrC(011), and CO_2_ and H to HCOO on TiC(111). The calculated data for those and all other reactions are reported in Table 3.

**Table 3:** Activation and Reactive Step Energies of CO_2_ Reduction over TiC and ZrC^a^

	*E*_ads_	*E*_a_	*E*_s_
TiC			
001—CO	–0.90	+2.13	–0.23
011—CO	–3.45	+0.69	–3.6
111—CO	–3.11	+0.99	–2.60
001—COOH	–1.84	+1.58	+0.59
011—COOH	–4.80	+2.15	+1.43
111—COOH	–4.19	+2.15	+1.43
001—HCOO	–1.84	N/A	+2.03
011—HCOO	–4.80	+1.39	+1.43
111—HCOO	–4.19	+1.44	+0.35
ZrC			
001—CO	–1.68	+1.86	–0.04
011—CO	–4.19	+0.49	–0.84
111—CO	–3.18	+1.29	–2.49
001—COOH	–2.65	+1.48	+0.95
011—COOH	–5.44	+1.97	+1.71
111—COOH	–4.38	+2.44	+1.91
001—HCOO	–2.65	N/A	+1.63
011—HCOO	–5.44	>3 eV	–0.48*
111—HCOO	–4.38	+1.58	+0.48

a All energies are referred to the adsorbed CO_2_, for dissociation reactions, or CO_2_ + H, for hydrogenation reactions, and are reported in eV. The first column refers to the adsorption energy; the second row is the activation energy, hence, the energy difference between the adsorbed reactant and the transition state; the final row reports the energy of the products referred to that of the adsorbed reactants.

Dissociation to CO on both (001) surfaces is mildly exothermic but shows very high barriers—over 2 eV for TiC(001). These results might seem inconsistent with the previous investigation of CO_2_ adsorption on the same surfaces,^15,28^ which highlight the efficient activation of the CO_2_ molecule on the (001) surfaces of several transition-metal carbides through the investigation of their elongated bond distances, modified bond angle, and effective charge transfer from the surface to the carbon center. The calculations presented here suggest that this discrepancy is due to an inefficient coordination of the transition state correlated to the dissociation of the C–O bond, since the electronic and geometric characteristics of adsorbed CO_2_ that indicate activation of the molecule^28^ do not translate into a sufficiently low activation barrier.

This result shows how, even if the (001) surface can chemisorb both reactants and products efficiently, CO_2_ is not sufficiently activated on these surfaces for appreciable dissociation to occur. CO_2_ adsorbed on TiC(001) and ZrC(001) shows longer bond lengths, a bent geometry, and a higher number of electrons than the gas-phase molecule, all characteristics that have been correlated with CO_2_ activation.^29^ However, this activation is ineffective for this reaction since on both carbides, the transition state has a higher energy than the gas-phase reactants, effectively ruling out the direct dissociation of CO_2_ on (001) surfaces.

The dissociation reaction is considerably more exothermic on (011) and (111) surfaces, as the more exposed metal atoms can coordinate CO_2_ more efficiently while the C–O bond is elongated until full dissociation. Dissociation on ZrC(011) is, of all reactions, the one with the lowest energetic barrier (*E*_a_ = 0.49 eV over the adsorbed reactants), while TiC(011) shows the most exothermic reaction energy (*E*_s_ = −3.60 eV) but has a slightly higher activation barrier, *E*_a_ = 0.69 eV. This can be correlated with the higher metal area exposed on ZrC, allowing for a better coordination of the dissociating oxygen but resulting in its less efficient coordination once the reaction is complete. However, this trend is inverted for the transition states on (111) surfaces: as a result of the larger cell parameters of ZrC, the dissociating oxygen cannot be coordinated as efficiently, resulting in a higher barrier than that on TiC(111).

Conversely, the hydrogenation reaction to carboxyl is most favorable on the (001) surfaces of TiC and ZrC. In all cases, the reaction step is endothermic, and the barrier is at least 1.5 eV above the adsorbed state of the reactants. However, this energy can be provided by the adsorption energies of CO_2_ and H_2_ on the surfaces of the catalysts, as both are significantly larger than the activation barriers. This reaction is thermodynamically least unfavorable on TiC(001), where it shows an *E*_s_ = +0.59 eV, while kinetically ZrC shows a slightly lower barrier of *E*_a_ = +1.48 eV. The hydrogenation on both (011) and (111) surfaces is instead significantly endothermic and shows barriers of 2 eV and higher, hindering the formation of COOH on these four surfaces. As mentioned in the previous section, the strong bond formed by hydrogen with these surfaces can be considered as a reason for the high hydrogenation barriers found on this surface.

The hydrogenation to formate presents an interesting case study, as the results vary greatly depending on the surface. On the (001) surfaces, as mentioned in the previous section, the reaction is highly endothermic and no investigation of the potential energy surface has been performed. On the two (011) surfaces, formate can dissociate rapidly to HCO + O. The process seems to be spontaneous on ZrC, for which no HCOO intermediate could be isolated, while it requires a small activation energy on TiC. This reaction is more viable than the hydrogenation to carboxyl despite its identical reaction energy, as *E*_a_(HCOO) = 1.47 eV < *E*_a_(COOH) = 2.44 eV. On ZrC(011), however, the structure of the transition state leading to the simultaneous hydrogenation and dissociation is found to be very close to the unstable intermediate HCOO, which obviously leads to a high kinetic barrier (*E*_a_ > 3 eV) after which the intermediate spontaneously dissociates. The height of this barrier rules out the possibility of hydrogenation to formate on the latter surface, as it is significantly higher than *E*_a_ = 1.97 eV found for carboxyl production. Conversely, on both (111) surfaces the reduction to formate is mildly endothermic, with *E*_s_ = 0.35 eV on TiC, probably due to the geometry of the surfaces, which allows for strong interaction of the carbon and oxygen atoms of HCOO with the metal atoms of the material while minimizing interaction with hydrogen. Activation barriers on the two surfaces are similar—*E*_a_(TiC) = 1.44 eV, *E*_a_(ZrC) = 1.58 eV. While the kinetic barrier to hydrogenation is high, it is still much lower than the adsorption energies of the reactants, CO_2_ and H_2_; it should therefore be possible to observe the COOH intermediate if the two reaction steps take place in such a way that the energy required to overcome the barrier to hydrogenation is provided by the adsorption onto the (111) surfaces.

### Surface-Mediated Product Selectivity

**Figure 4:**
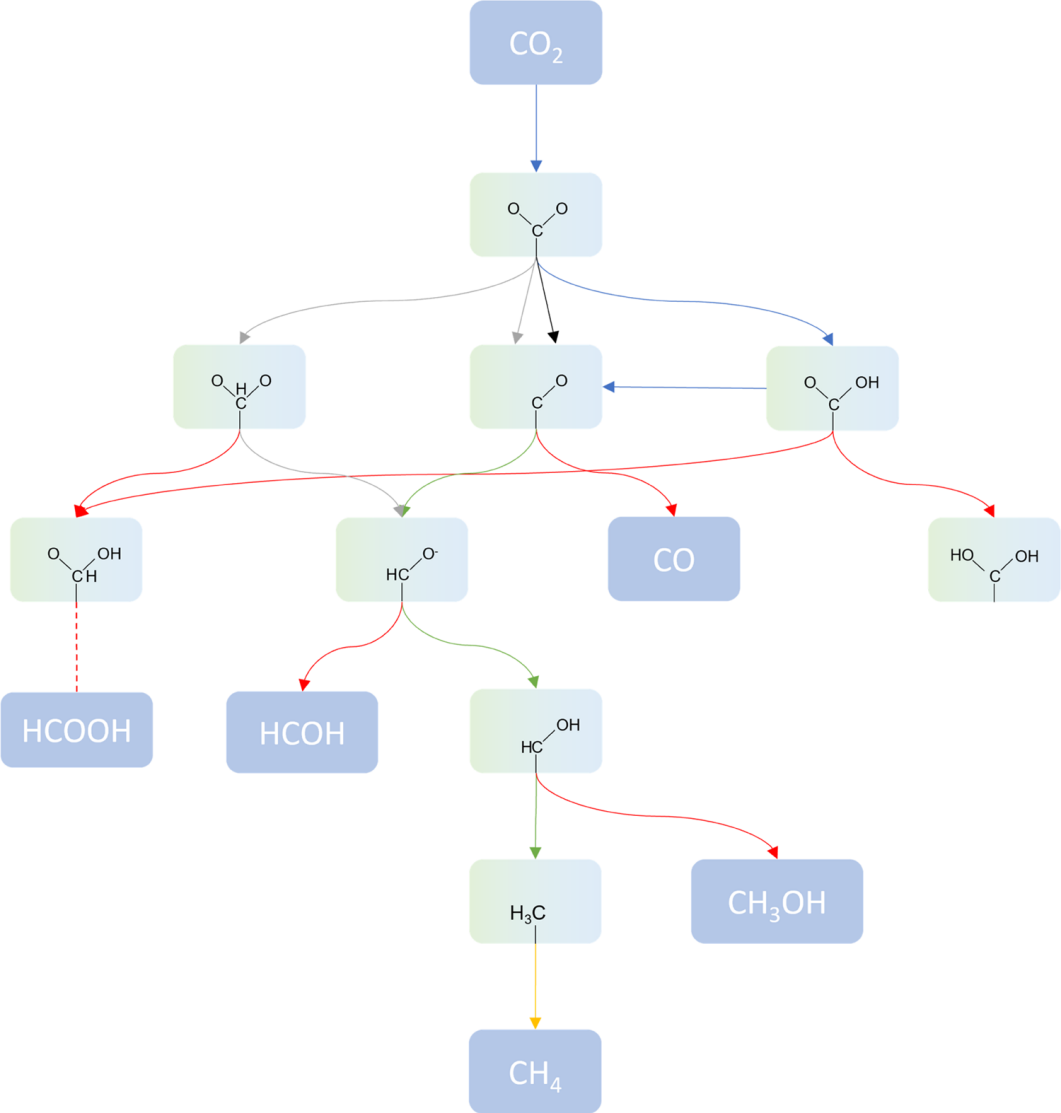
Proposed reduction pathways of CO_2_ on transition-metal carbide surfaces. The background chosen for each compound corresponds to its phase: dark blue for gas phase, and green and light blue for molecules on TiC and ZrC, respectively. The color code of the arrows between each compound is associated with the results of the present investigation: blue for reactions favored on (001) surfaces, black for (011), gray for (111), green for reactions favored on all surfaces, red for reactions hindered by either thermodynamic or kinetic impediments, and yellow for desorption reactions. As will be discussed in this paper, the main reduction path on all surfaces is that leading to CH_4_ through a HCO intermediate.

After the first reduction step, multiple pathways are available for the conversion of CO_2_ into useful chemicals, as illustrated in Figure 4. The results highlighted in the first part of the present work allowed us to reduce the number of investigated surfaces for the subsequent reaction steps by excluding those involving high activation energies. The final part of our study will investigate, first, the reaction steps following the dissociation to CO on the (011) and (111) surfaces, and second, those following the hydrogenation to COOH on the (001) and HCOO on the (111) surfaces. All other reaction paths, as discussed above, have been discounted due to the high energies of their intermediate steps or transition states, which inhibit further reaction.

The investigation of the second steps of the reaction has entailed the analysis of the thermodynamic reaction energies of the pathways for the conversion of COOH and HCOO to HCOOH on the (001) and (111) surfaces, respectively; COOH to CO on (001) surfaces; and CO to HCO and COH on (011) and (111) surfaces. On the (111) surface of TiC, all attempts at optimizing HCOOH led to its decomposition to HCO and OH. It was therefore concluded that all reactivity would proceed via HCO rather than via HCOOH. The alternative ZrC(111) facet does enable the formation of a stable HCOOH intermediate. However, despite the negative reaction energy of *E*_r_ = −0.51 eV, this step is strongly endothermic, with *E*_s_ = 3.4 eV with respect to HCOO. Such an endothermic reaction energy will be inaccessible, and therefore formic acid is also ruled out as a final product on the ZrC(111) surface.

**Figure 5:**
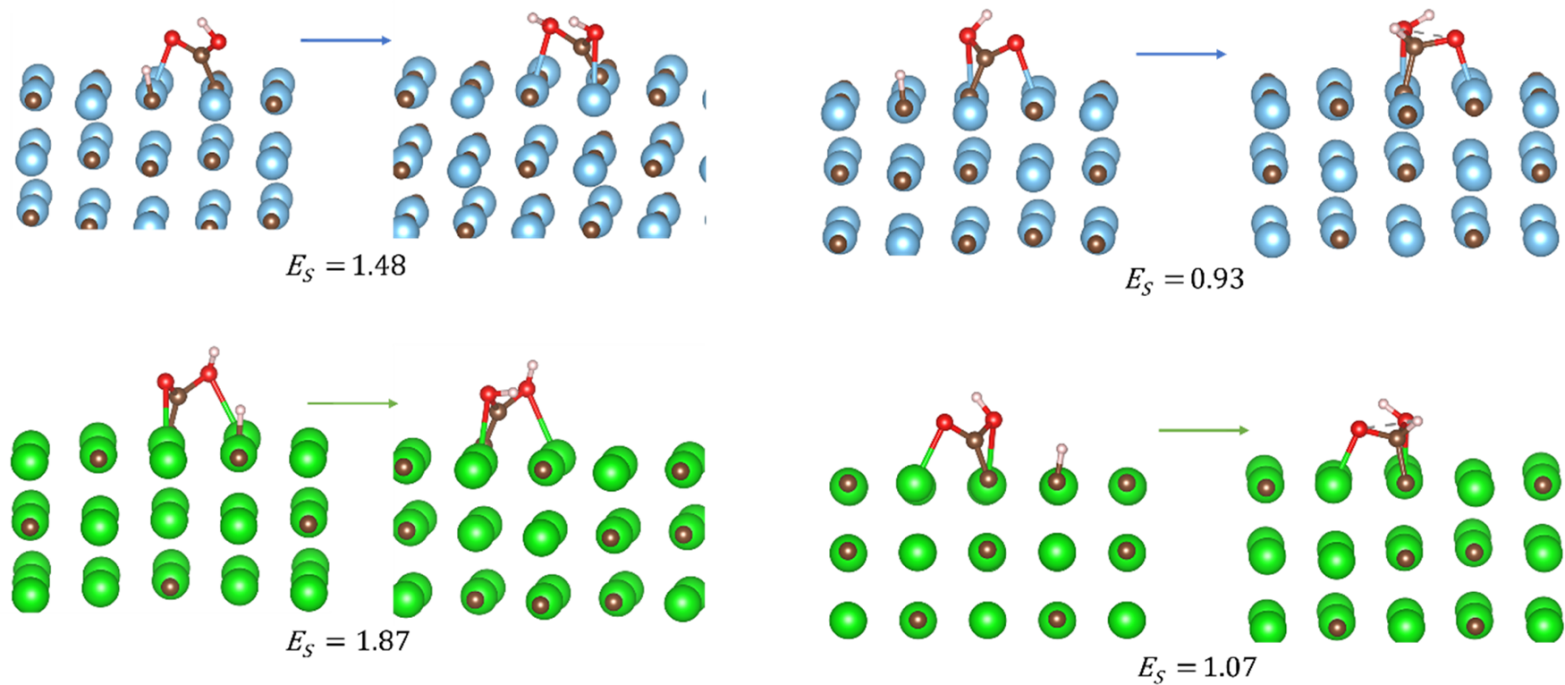
TiC (top) and ZrC (bottom) (001) surface-mediated formation of dihydroxymethylidene (left) and formic acid (right). All energies are given in eV.

To assess the viability of COOH hydrogenation on the (001) facets of TiC and ZrC, the reaction landscapes for the transfer of a surface hydrogen to either oxygen atom or to the carbon center were computed. Figure 5 shows the driving force for the production of dihydroxymethylidene and formic acid from COOH on the (001) surfaces of TiC and ZrC. The reaction energy for the formation of dihydroxymethylidene is far too high for it to be an important intermediate in the further reduction of COOH and was not considered further. Similarly, a second hydrogenation of the hydroxyl oxygen followed by the dissociation of the product into CO and H_2_O is shown to be highly endothermic, with *E*_s_ = 1.67 eV on TiC(001) and *E*_s_ = 1.99 eV on ZrC(001).

While still endothermic, the reaction energy for formic acid formation (∼1 eV) appears to be more accessible, so CI-NEB calculations were undertaken to determine the activation energies for such a reaction. Unfortunately, the results showed that these pathways were kinetically blocked by barriers larger than 2 eV. The direct formation of formic acid was therefore ruled out as a possible outcome of the CO_2_ reduction upon these catalysts. As a result, the investigation shifted toward the hydroxylation of surface-bound CO/HCO intermediates, as the most likely reaction paths for carbide-catalyzed CO_2_ reduction.

**Figure 6:**
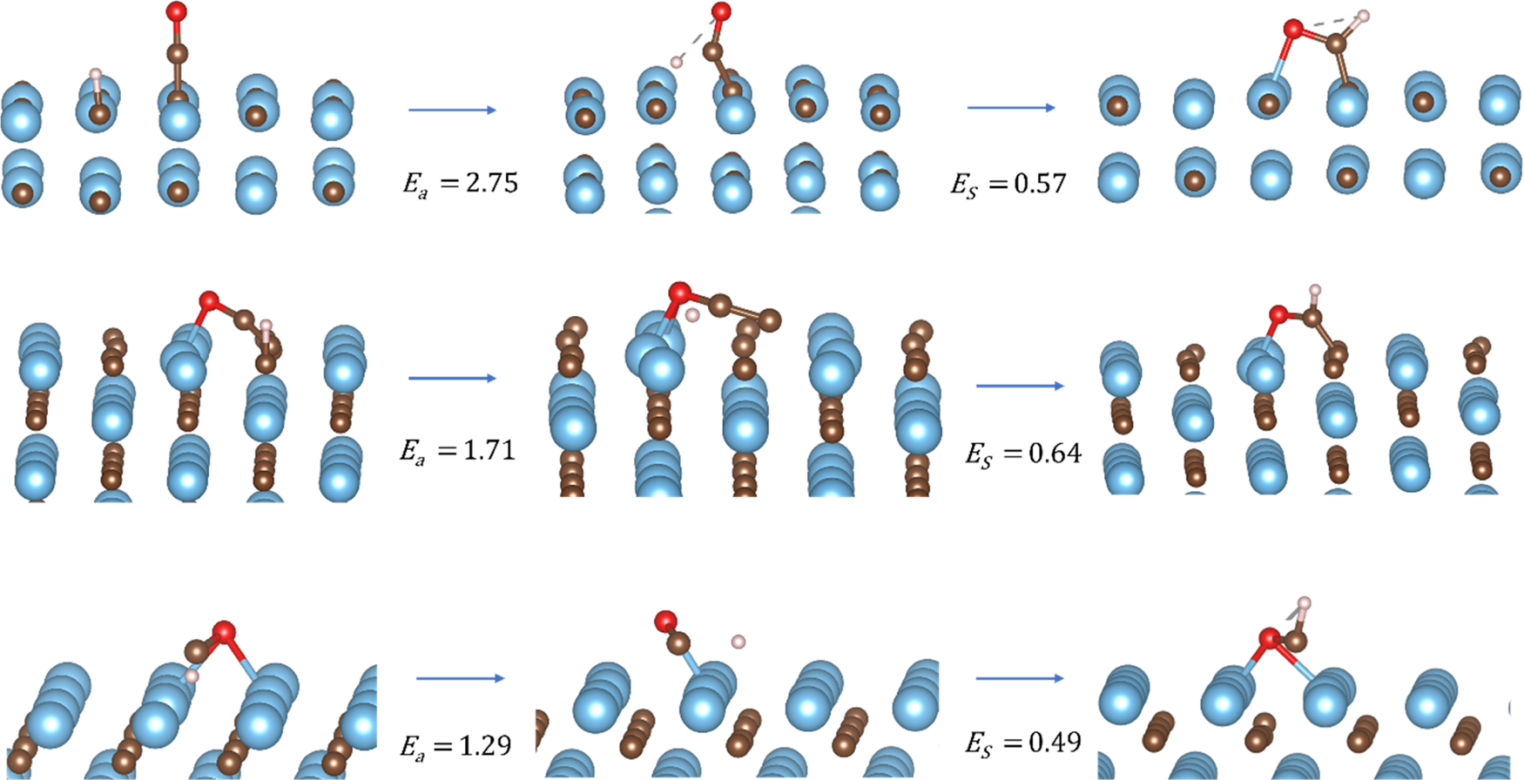
Surface-mediated HCO formation by TiC by (top) (001), (middle) (011), and (bottom) (111)-metal-terminated facets. All energies are given in eV related to surface-bound carbon monoxide and hydrogen.

Our calculations suggest that the most likely route for the CO/HCO intermediates on both (001) facets was the surface-mediated decomposition of COOH to CO and OH. It was determined that such a decomposition corresponds to a decrease in energy of *E*_s_ = −0.70 eV on the ZrC(001) facet but was slightly endothermic on the TiC(001) surface (*E*_s_ = +0.21 eV). Nevertheless, both reaction steps can be considered accessible when considering both the potential gain in entropy as COOH is dissociated and the inherent margin of error of the calculated energies. Therefore, it might be expected that COOH readily decomposes on the (001) termination of ZrC and COOH/CO + OH forms an equilibrium on the (001) surface of TiC, which is then pushed toward the decomposed product as CO goes through further reduction. For completeness, the decomposition of formate (to HCO + O) was also considered, obtaining reactive step energies of *E*_s_ = +0.46 eV and *E*_s_ = +1.02 eV on the (001) surfaces of TiC and ZrC, respectively. Calculations discussed below examining CO hydrogenation will indicate that the decomposition of formate might be important as a shortcut to HCO formation on the (001) surface of TiC (see Figure 6). However, carboxylic acid decomposition is likely to be the dominant pathway for the alternative ZrC(001) facet.

**Figure 7:**
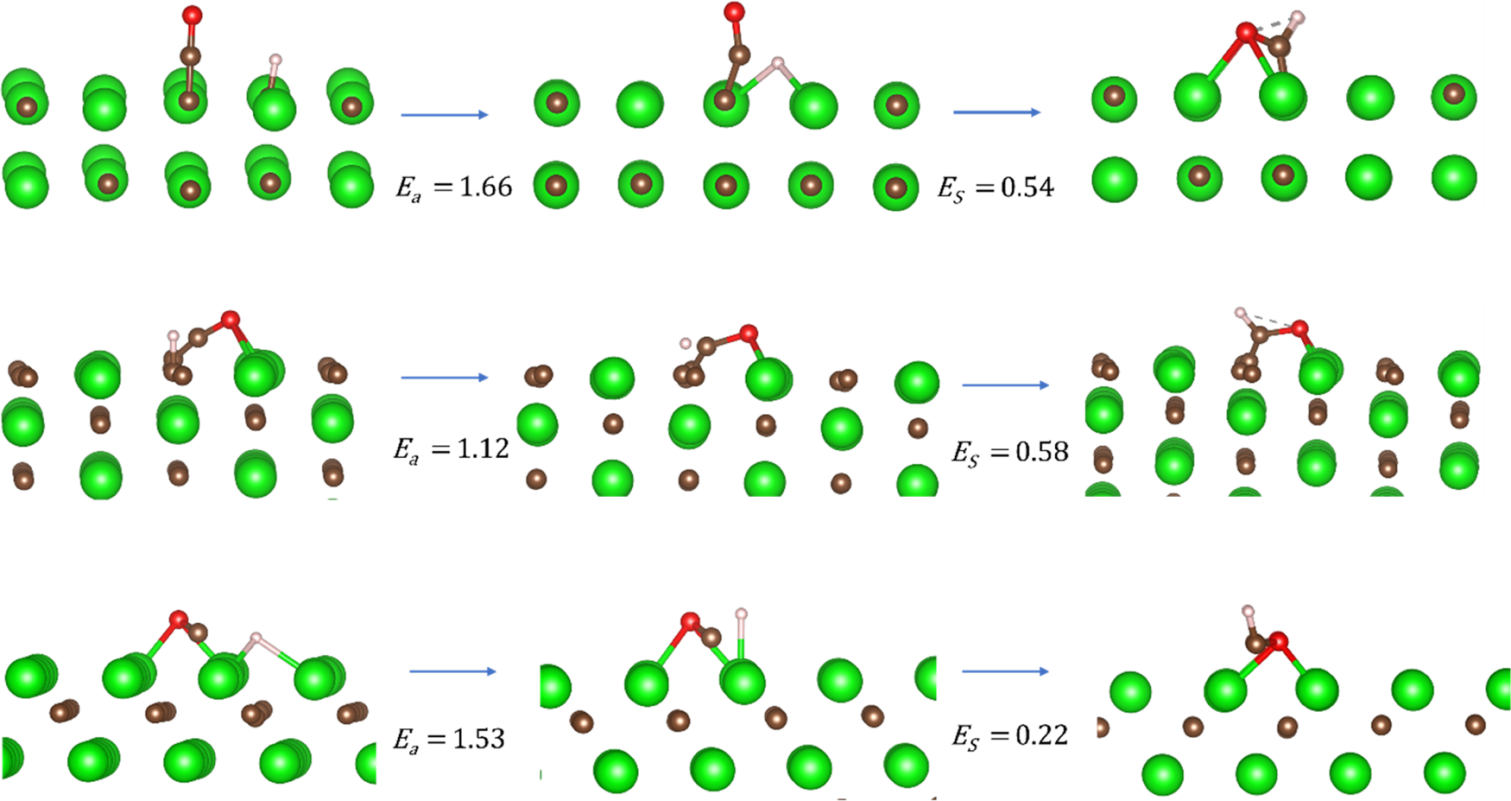
Surface-mediated HCO formation by ZrC by (top) (001), (middle) (011), and (bottom) (111)-metal-terminated facets. All energies are given in eV related to surface-bound carbon monoxide and hydrogen.

Data from Figure 6 show that the most active surface for carbon monoxide hydrogenation to HCO on TiC is the metal-terminated (111) surface, followed closely by the (011) facet. While this ordering is reversed on ZrC, all barriers were assessed to be larger than 1 eV. As mentioned above, an activation barrier of almost 3 eV, such as that found for HCO formation on TiC(001), clearly implicates formate decomposition as a more likely mechanism for HCO formation than CO hydrogenation. Interestingly, these results indicate that surface geometry—and especially CO adsorption morphology—plays a far more significant factor in the activation energies for CO hydrogenation than does hydrogen adsorption, with no obvious correlation between barrier heights and the surface hydrogenation energies previously reported using comparable methodologies.^16^ There are far more product-like and higher-energy transition states on the two (001) surfaces, and the fact that the preferred binding site for the hydrogen and carbon atoms are both located on top of a surface carbon site implies that these late transition states are far from optimal. The more favorable barrier seen on the (111) surface of TiC over the comparable facet of ZrC is also due to surface morphology, as the shorter TiC lattice constants enable a transition state where the hydrogen atom is close to a highly coordinated hollow site. The hydrogen position in the comparable ZrC(111) transition state, on the other hand, more closely resembles a C_on-top_ low-coordinated position, as shown in Figure 7.

Across all surfaces investigated, our calculations have indicated the importance of a common HCO intermediate, either as a result of direct hydrogenation of CO (as is the case for (011) and (111) facets) or via the (001) surface-mediated decomposition of formate or carboxylic acid. It was therefore decided to focus on the further reduction of HCO. Table 4 shows the reaction energies for HCO hydrogenation to formaldehyde. Crucially, the desorption energy for formaldehyde is very high for all surfaces except for TiC(111), where the value for H_2_CO release is actually slightly negative (−0.09 eV/mol). Additionally, when examining the transition-state energies of formaldehyde formation, we can rule out that this process takes place on the TiC(111) surface entirely. On this surface, the alternative and highly exothermic oxygen hydrogenation step appears far more likely, but no stable HCOH minima could be found on either (011) surface, with HCOH undergoing a barrierless decomposition to surface-bound HC and OH species.

**Table 4:** Activation and Reaction Energies of HCO Reduction over TiC and ZrC^a^

	*E*_ads_(HCO)	*E*_a_	*E*_s_
TiC			
001—H_2_CO	–1.81	+1.67	–1.49
011—H_2_CO	–4.11	+1.77	–6.78
111—H_2_CO	–3.88	+5.60	–0.49
001—HCOH	–1.81	+1.08	–2.12
011—HC OH^b^	–4.11		–5.80
111—HC OH^b^	–3.88		–5.26
ZrC			
001—H_2_CO	–2.25	+5.86	–2.01
011—H_2_CO	–4.57	+1.70	–4.51
111—H_2_CO	–3.89	+1.43	–3.93
001—HCOH	–2.25	+1.80	–1.89
011—HC OH^b^	–4.57		–5.83
111—HCOH	–3.89	+2.63	–2.85

a The first column refers to the surface-bound HCO with reference to gas-phase CO and one-half of H_2_*E*_ads_(HCO); the second row is the activation energy *E*_a_, and the third and final row reports the energy of the products referred to that of the reactants *E*_s_. All energies are reported in eV.

The decomposition of HCOH is likely to promote the production of various surface-bound C1 hydrocarbons and water, with no obvious route through to methanol formation. However, the strongly bound formaldehyde on the other surfaces does offer the potential for the production of the alcohol product. Therefore, the hydrogenation of H_2_CO was examined on all surfaces, with the resulting reaction energies given in Table 5. Hydrogenation of formaldehyde to CH_3_O is an essentially thermodynamically neutral process on both (001) surfaces and on the (011) facet of ZrC, while the reaction energy is highly exothermic on both (111) surfaces studied. Importantly, the adsorption energy for surface-bound formaldehyde is far too large to enable the desorption to occur on any of these facets. Interestingly, the reaction energy is highly endothermic on the (011) facet of TiC, which is due to the bidentate binding of formaldehyde causing an additional σ-bond not seen in the monodentate H_3_CO. Most importantly, and despite repeated attempts, no stable methanol species could be produced by hydrogenation of surface-bound H_3_CO, and in all cases, decomposition to surface CH_3_ and OH species was observed to be a barrierless process. This result opens a new route toward methane and water formation, with the alternative pathway to methanol being again blocked by the surface-mediated cleavage of the C–O bond.

**Table 5:** Reaction Energies *E*_r_ for H_2_CO Reduction to H_3_CO and H_3_COH over TiC and ZrC^a^

	H_2_CO	H_3_CO	H_3_C + OH
TiC			
001—H_3_C OH	–1.49	–1.74	–2.92
011—H_3_C OH	–6.78	–3.98	–8.11
111—H_3_C OH	–0.49	–4.78	–6.63
ZrC			
001—H_3_C OH	–2.01	–1.94	–3.33
011—H_3_C OH	–4.51	–4.44	–6.39
111—H_3_C OH	–3.93	–4.87	–6.17

a Due to the lack of a stable CH_3_OH intermediate, the last column refers to adsorbed CH_3_ and OH species on each surface. All energies are reported in eV.

**Figure 8:**
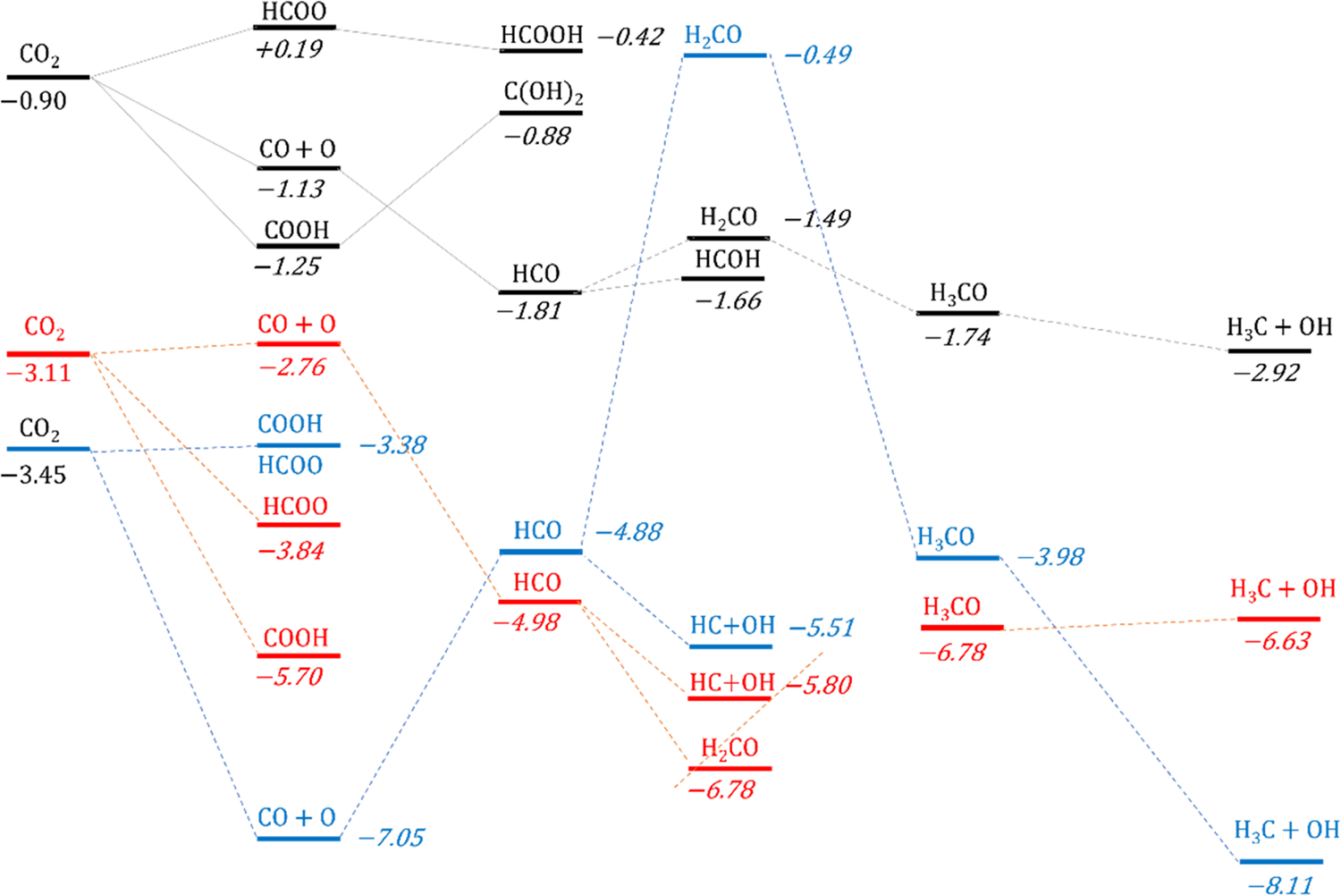
Thermodynamic profile of the reaction paths for CO_2_ reduction on TiC (001) (black), (011) (blue), and (111) (red) surfaces. The numbers displayed are the reaction energies of each intermediate (*E*_s_), in eV. All energies are referred to all reactants and products being simultaneously adsorbed on the relevant surfaces, without interaction between the adsorbed species, so that the reference energy level corresponds to two adsorbed hydrogen molecules and gas-phase CO_2_ for all steps. Only the carbon-containing molecule of each step is reported for clarity.

**Figure 9:**
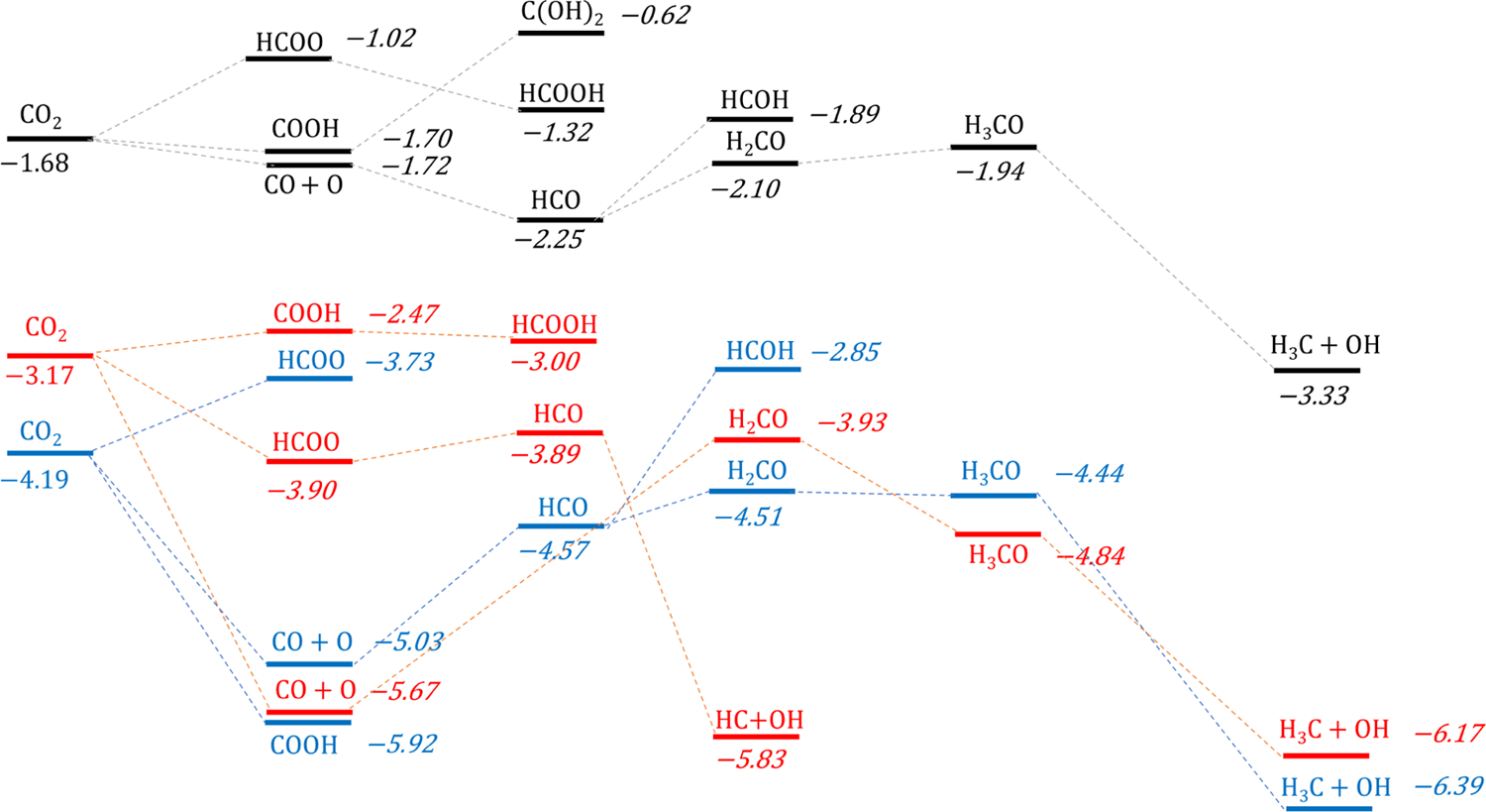
Thermodynamic profile of the reduction paths for CO_2_ reduction on ZrC (001) (black), (011) (blue), and (111) (red) surfaces. The numbers displayed are the reaction energies of each intermediate (*E*_s_), in eV. All energies are referred to all reactants and products being simultaneously adsorbed on the relevant surfaces, without interaction between the adsorbed species, so that the reference energy level corresponds to two adsorbed hydrogen molecules and gas-phase CO_2_ for all steps. Only the carbon-containing molecule of each step is reported for clarity.

Taken together, these results help to rationalize the observed selectivity toward methane production over methanol when CO_2_ conversion by TiC and ZrC is investigated.^10,30^ The selectivity that these materials have demonstrated experimentally is here confirmed computationally on all low-index surfaces, as all paths leading to CO and other valuable products are blocked by either endothermic reactions or very high activation energies. Furthermore, the match between experimental and computational results suggests the importance of the HCO intermediate on all facets of ZrC and TiC as a fundamental step in the reduction path for CO_2_. The color scheme of Figure 4 shows the favorable and unfavorable reactions on each surface, rationalizing the product selectivity and proposing a few possible reduction paths. These results provide a clearer picture of the reactivity on each facet of the carbides. Figures 8 and 9 combine the reduction paths of CO_2_ on the three surfaces of TiC and ZrC, displaying the reaction energies (i.e., the energy of the adsorbed intermediate relative to the appropriate number of CO_2_ and H_2_ molecules in the gas phase) of each intermediate along the six paths. The reaction landscapes are similar for the two carbides, further revealing the similarity between the catalytic properties of the two materials, and in all cases, the HCO intermediate is central within the reduction path to CH_4_. Furthermore, on both materials, the (001) surface presents the least negative energies, suggesting an easier release of CH_4_ and a consequently better catalytic activity for these facets, in contrast to previous reports on the hydrogen evolution reaction on the same surfaces,^16^ for which the (001) facet appeared to be too stable to catalyze the reaction adequately. On both carbides, however, reported conversion rates are low, probably due to oxidation of the surface under reaction conditions.^10,30^ This behavior might be due to the highly negative reaction energies reported in Figures 8 and 9 (−3 to −8 eV), which slow the release of the reduced products, as well as to the strong adsorption of oxygen on the surface, reported in Table 1, which might create a passivating layer at the interface of the catalyst.

## Summary and Conclusions

This work has reported a systematic study into the selective hydrogenation of carbon dioxide over the low-Miller index (001), (011), and (111) surfaces of TiC and ZrC. DFT calculations and periodic models have been used to assess the viability of reaction mechanisms toward formic acid, carbon monoxide, formaldehyde, methanol, and methane. Carbon dioxide adsorbs very exothermically on all surfaces and is highly reduced (in many cases by the transfer of more than one electron from the carbide), with the highly activated adsorbate readily undergoing further surface-mediated reactions. The C–O bond cleavage appears to be favored on both (011) surfaces, while COOH formation appears to be the initial step on the two (001) facets. The alternate metal-terminated (111) surfaces are capable of catalyzing both carbon monoxide and formate production as their primary reaction step. Whichever initial mechanism is promoted, all accessible reduction pathways go through a HCO intermediate and ultimately promote the formation of methane over any other reduction product. These results help to rationalize the experimentally observed selectivity toward methane over carbon monoxide when CO_2_ conversion by hydrogen is studied and further demonstrate the difference in chemical reactivity of the three facets of each carbide.

## Supporting Information Available

Activation energy for CO_2_ adsorption reported in this work compared to previously reported values^15^ (Table S1) (PDF)

## Notes

All data created as part of this study are openly available at: http://doi.org/10.17035/d.2022.0162515110.

## Abbreviations

DFT: density functional theory
PBE: Perdew–Burke–Ernzerhof
PAW: projected-augmented wave
TMC: transition-metal carbide
