# Supplementary material for: Catalytic Reduction of Carbon Dioxide on the (001), (011), and (111) Surfaces of TiC and ZrC: A Computational Study
Source: J Phys Chem C Nanomater Interfaces. 2022 Mar 14;126(11):5138–50. doi: 10.1021/acs.jpcc.1c10180 (PMC8958596; doi:10.1021/acs.jpcc.1c10180)
Supplement: Supplementary file 1 — jp1c10180_si_001.pdf [file jp1c10180_si_001.pdf]

Supporting information

The Catalytic Reduction of Carbon Dioxide on the (001), (011), and (111) surfaces of TiC and ZrC: a Computational Study

Fabrizio Silveri[1,2,3], Matthew G. Quesne[1,4] Francesc Viñes Solana[2], Francesc Illas Riera[2], C. Richard A. Catlow[1,4,6] and Nora H. de Leeuw[1,5]*

[1]School of Chemistry, Cardiff University, Main Building, Park Place, Cardiff CF10 3AT, UK.

[2]Departament de Ciència de Materials i Química Física and Institut de Química Teòrica i Computacional (IQTCUB), Universitat de Barcelona, c/Martí i Franquès 1-11, 08028 Barcelona, Spain

[3]Gemmate Technologies s.r.l., via Reano, 31, 10090 Buttigliera Alta (TO), Italy

[4]UK Catalysis Hub, Research Complex at Harwell, STFC Rutherford Appleton Laboratory, Didcot, Oxfordshire OX11 0FA, UK

[5]School of Chemistry, University of Leeds, Leeds LS2 9JT, UK

[6]Department of Chemistry, University College London, 20 Gordon St., London WC1 HOAJ, UK

Table S1 Activation energy for CO_2 adsorption reported in this work compared to previously reported values[7].

	TiC			ZrC		
	$^{a}E_{r}$	$^{b}E_{r}$	*%diff*	$^{a}E_{r}$	$^{b}E_{r}$	*%diff*
**(001)**	-0.90	-0.86	-0.05	-1.68	-1.62	-0.04
**(011)**	-3.45	-3.45	0.00	-4.19	-1.03	-3.07
**(111)**	-3.11	-3.05	-0.02	-3.17	-3.32	0.04

$^{a}E_{r}$ lowest energy CO_2 adsorption energy. $^{b}E_{r}$ previously reported CO_2 adsorption values using the geometry scan method[1]. ***%diff*** percentage between CO2 adsorption values determined using both methods.

References

(1) Quesne, M. G.; Roldan, A.; De Leeuw, N. H.; Catlow, C. R. A. Carbon Dioxide and Water Co-Adsorption on the Low-Index Surfaces of TiC, VC, ZrC and NbC: A DFT Study. *Phys. Chem. Chem. Phys.* **2019**, *21* (20). https://doi.org/10.1039/c9cp00924h.
